# The importance of mother-child interaction on smart device usage and behavior outcomes among toddlers: a longitudinal study

**DOI:** 10.1186/s13034-024-00772-6

**Published:** 2024-06-28

**Authors:** Pairote Chakranon, Jian-Pei Huang, Heng-Kien Au, Chen-Li Lin, Yi-Yung Chen, Shih-Peng Mao, Wen-Yi Lin, Ming-Lun Zou, Wanda Estinfort, Yi-Hua Chen

**Affiliations:** 1 School of Public Health, College of Public Health, Taipei Medical University, New Taipei City, Taiwan; 2 Department of Obstetrics and Gynecology, Mackay Memorial Hospital, Taipei, Taiwan; 3 Department of Obstetrics and Gynecology, Taipei Medical University Hospital, Taipei, Taiwan; 4 Department of Obstetrics and Gynecology, School of Medicine, College of Medicine, Taipei Medical University, Taipei, Taiwan; 5 Department of Obstetrics and Gynecology, Heping Fuyou Branch, Taipei City Hospital, Taipei, Taiwan; 6 Department of Obstetrics and Gynecology, Ministry of Health and Welfare, Taipei Medical University-Shuang Ho Hospital, New Taipei City, Taiwan; 7 Ph.D. Program in Medical Neuroscience, College of Medical Science and Technology, Taipei Medical University and National Health Research Institutes, New Taipei City, Taiwan; 8 Department of Epidemiology and Environmental Health, School of Public Health and Health Professions, University at Buffalo, State University of New York, Buffalo, USA; 9 Neuroscience Research Center, Taipei Medical University, Taipei, Taiwan; 10 Research Center of Health Equity, College of Public Health, Taipei Medical University, New Taipei City, Taiwan; 11 School of Public Health, College of Public Health, Taipei Medical University, 10F., Biomedical Technology Building, Shuang-Ho Campus, No. 301, Yuantong Rd., Zhonghe Dist., New Taipei City, Taiwan

**Keywords:** Early smart device use, Media content, Behavioral problem, Mother–child interaction, Toddler

## Abstract

**Background:**

In recent years, smart devices have become an integral part of daily life. However, longitudinal studies, particularly those regarding the relationship between toddlers’ smart device usage and behavioral outcomes, are limited. Understanding the impact of parent–child interactions on this relationship is crucial for enhancing toddlers’ developmental outcomes. Accordingly, this study examined the influence of early screen time and media content exposure on toddlers’ behaviors, as well as the positive effects of mother–child interactions on this influence.

**Methods:**

We used relevant data related to 277 children born between November 2016 and July 2020 and who were part of an ongoing prospective follow-up study conducted across five hospitals in Taipei City, Taiwan. We analyzed (1) data from maternal reports regarding children’s behavior by using the Child Behavior Checklist (for ages 1^1/2^–5 years), (2) assessments of mother–child interactions by using the Brigance Parent–Child Interactions Scale, and (3) self-reported parental data covering the first 3 postpartum years. Statistical analyses involved group-based trajectory modeling and multiple linear regression.

**Results:**

A considerable increase in screen time between the ages of 1 and 3 years was associated with less favorable behavioral outcomes at age 3. These outcomes included somatic complaints [adjusted beta coefficient (aβ) = 2.17, 95% confidence interval (CI) = 0.39–3.95, p-value = 0.01], withdrawal (aβ = 2.42, 95% CI = 0.15–4.69, p-value = 0.04), and aggressive behavior (aβ = 6.53, 95% CI = 0.25–12.81, p-value = 0.04). This association was particularly evident among children with lower levels of mother–child interaction. Nevertheless, positive mother–child interactions mitigated most of the adverse effects. Additionally, increased exposure to games and cartoons was associated with poorer behavioral outcomes in all children except for those experiencing positive mother–child interactions.

**Conclusion:**

Early mother–child interactions play a crucial role in mitigating the risk of behavioral problems in toddlers who spend prolonged periods looking at screens and who are frequently exposed to game and cartoon content.

**Supplementary Information:**

The online version contains supplementary material available at 10.1186/s13034-024-00772-6.

## Introduction

In the present technologically advanced era, smart devices such as smartphones and tablets are becoming increasingly integrated into the daily lives of individuals across all age groups, including toddlers [1]. Given the pivotal role of the toddler years in brain development and the establishment of lifelong habits [2, 3], the American Academy of Pediatrics proposed the following recommendations: no screen exposure except for video chats for children aged < 2 years and a 1-hour daily limit on such exposure for children aged 2–5 years [4]. These recommendations are in line with the corresponding World Health Organization guidelines [5]. However, a meta-analysis reported that a minority of children, approximately 25% of those aged under 2 years and 36% of those aged 2 to 5 years, adhered to these recommended screen time limits [6]. Considering the potential impact of screen time on the cognitive, behavioral, and social development of children in the long term, the development of preventive strategies that can be implemented from early childhood is imperative.

Increased screen time in young children has been associated with a range of behavioral problems, including hyperactivity [7], inattention [8], emotional and behavioral dysregulation [9–12], and relatively low overall well-being [13]. Such increased exposure may also have detrimental effects on children’s long-term development, affecting their behavior, social interactions, and academic performance [14, 15]. However, a notable gap in the literature exists regarding the distinction between the effects of traditional media and those of smart devices, with only two studies having focused on smart device screen time among toddlers. One such study, conducted by Lin et al. [16], focused on toddlers aged 18–36 months by using the Child Behavior Checklist (CBCL) to assess behavioral performance. Their findings indicated a correlation between increased time spent using touch screen devices and heightened emotional difficulties and aggression. In another study conducted by Levine et al. [17] and involving children aged under 36 months, mobile media use was associated with noneducational parental motivations in children exhibiting higher levels of self-regulation difficulties. However, notably, the cross-sectional designs of those studies limited their capacity to observe changes over time.

In addition to duration of screen time, the specific applications and media content accessed on smart devices also play crucial roles [4]. Radesky et al. [18] revealed that children predominantly consume media from video-sharing platforms such as YouTube, with only approximately one quarter of such content being educational. Different types of media content can have different developmental effects on children. For instance, younger children viewing educational content and engaging in video chats may receive benefits [19, 20], whereas exposure to age-inappropriate or violent content, for example, can be detrimental [21]. However, positive mother–child interactions, which reflect maternal sensitivity and responsiveness and thus are crucial for child development, may help mitigate the adverse effects of screen time [22, 23]. A nationally representative study conducted in the United States demonstrated that warm and responsive parenting moderated the relationship between exposure to background television and executive functioning in early childhood [24]. Positive parenting practices stemming from constructive interactions can guide children toward beneficial smart device usage, curb excessive screen time, and encourage engagement with more suitable media content to promote positive child development. These findings underscore the importance of positive interactions and attentive parenting in regulating children’s media consumption [25].

Despite the emphasis on the association between screen time and child development, longitudinal studies specifically focused on the behavioral effects of smart device usage on toddlers are scarce. Nevertheless, many studies have examined the impact of a combination of traditional media (e.g., television) and smart media [7–13]; notably, television offers passive viewing, whereas smart devices provide interactive experiences along with heightened auditory and visual stimulation through personalized, hands-on engagement. Given these differences, an investigation into the impact of smart device usage on children’s developmental outcomes is paramount. Smart devices pose challenges related to monitoring children’s activities, including the content that they access and the timing and location of media usage [1]. Such devices are frequently employed as distractions or rewards or to provide parental relief [26], which can exacerbate their negative effects. Moreover, the emission of blue light from smart devices can disrupt sleep patterns, potentially affecting a child’s health and development, particularly when used shortly before bedtime [27, 28].

The behavioral performance of young children is considerably influenced by parenting practices, with mother–child interactions playing a pivotal role in shaping parenting quality and its effects. Although no study has specifically addressed smart device usage, one study highlighted the potential of parent–child interactions to mitigate the risks associated with exposure to background television and executive function in high-risk children; the same study revealed that exposure to educational television serves as a protective factor for such children [24]. Considering the inevitable integration of smart devices into daily life in the present day, the potential impact of such devices, particularly with respect to health and developmental concerns for the vulnerable toddler population, must be emphasized. Accordingly, the present study investigated the role of mother–child interactions, the effects of a diverse range of media content formats, and the impact of frequent usage of smart devices on toddlers’ developmental outcomes.

To address gaps in the literature, the present study investigated the impact of screen time and media content exposure during the ages of 1–3 years on children’s behavioral performance at 3 years of age. Moreover, the potential modifying effects of mother–child interactions on this association was explored given that such effects have yet to be explored in longitudinal studies. Furthermore, we considered potential confounders identified in previous studies, including parental age, maternal education, maternal depression, parity, and the child’s sex [24, 25, 29, 30], along with pregnancy outcomes including preterm birth and low birthweight status [16, 31].

We hypothesized that a substantial increase in screen usage, coupled with exposure to game and cartoon content, between the ages of 1 and 3 years would be associated with an increase in behavioral problems in children aged 3 years. Furthermore, we posited that this effect would be ameliorated in children who experience positive mother–child interactions.

## Methods

### Study design and sample

In this study, we used data collected by the Longitudinal Examination Across Prenatal and Postpartum Health in Taiwan (LEAPP-HIT), an ongoing prospective study initiated in 2011 in Taipei, Taiwan. Pregnant women, along with their partners, were invited to participate in the present study during their early prenatal visits to outpatient clinics in five selected hospitals in Taipei (onsite baseline assessment). Subsequently, these women were followed up through postal surveys until the sixth postpartum year. To maximize the response rate, trained interviewers offered comprehensive explanations and telephone reminders. Specifically, we recruited pregnant women who were aged > 20 years, were in the early stages of pregnancy (< 16 weeks), intended to carry the pregnancy to full term, spoke Mandarin Chinese as their mother tongue, and had partners willing to participate in the present study (Supplementary Fig. S4).

Self-report instruments were used to evaluate the most crucial years of early development in humans. Data from the LEAPP-HIT project were collected during early pregnancy (i.e., before 16 gestational weeks, considered as baseline) and at four postnatal follow-up time points (i.e., at 1 month, 1 year, 2 years, and 3 years postpartum). Because we introduced the CBCL (1^1/2^-5; primary outcome) in November 2019, our sample included children born between November 2016 and July 2020, expected to complete the CBCL questionnaire at the age of 3 years between 2019 and 2023 (*n* = 506). Among these participants, 229 were lost to follow-up, leaving 277 participants remaining for the final analysis in this study. A comparison between the included and excluded participants revealed no significant differences in parental sociodemographic, birth outcome, or child characteristic variables (Supplementary Table S1 and Supplementary Fig. S1). Before interviews were conducted, written informed consent was obtained from all the participants. The study protocols were approved by the institutional review boards of all the study hospitals.

### Instruments and measures

#### Outcome variable: children’s behavioral problems

The CBCL is a widely recognized parental assessment tool designed to evaluate behavioral and emotional difficulties in children aged 1^1/2^-5 years in order to reflect their behavior over the preceding 2 months [32]. The CBCL, which comprises 99 items rated on a 3-point scale (not true, somewhat/sometimes true, and very true/often true) and 1 open-ended item, assesses behaviors across seven domains: emotional reactivity, anxiety/depression, somatic complaints, withdrawal, sleep problems, attention problems, and aggressive behavior. These domains are further categorized as “internalizing,” “externalizing,” and “total” problems. This study examined behavioral performance across seven domains of CBCL assessments, along with the three additional categories (internalizing, externalizing, and total problems). The CBCL has demonstrated high validity and reliability [33], including in Taiwan [16, 34].

#### Main independent variables

##### Time spent on smart devices by children

This study focused on households where mothers and children had access to smart devices. The mothers were instructed to periodically report on their child’s time spent on smart devices during the ages of 1–3 years, including both typical weekdays and weekends. These reports included responses to questions such as, “How long does your child usually spend on smart devices (e.g., smartphones and tablets) in total?” The daily mean time spent on smart devices (i.e., “screen time”) was calculated as [(time on weekday × 5) + (time on weekend × 2)]/7 [35], measured in minutes per day. This calculation method is consistent with those employed in many previous studies [12, 35]. Subjective reports from mothers are commonly employed in such studies, as demonstrated by a systematic review summarizing screen time measurement among young children aged 0–6 years [36].

##### Media content consumption of children

When their child was aged 3 years, each participating mother was asked to evaluate the type of content that their child consumed on smart devices. Related questions included inquiries such as, “How often does your child engage in primary activities—including engagement in education, cartoons, games, or voice or video calls—when using smart devices (e.g., smartphones and tablets)?” Responses to these questions were rated on a 5-point Likert scale ranging from 1 (*always*) to 5 (*never*). Each content variable was then dichotomized into two groups: higher use (*always* or *often*; 1) and lower use (*sometimes*, *seldom*, or *never*; 0). Owing to the small proportion of game content exposure, this variable was combined with cartoon content to create a combined category of game and cartoon content.

#### Moderator: mother–child interaction

This study routinely assessed mother-reported interactions between mothers and their children aged 1–3 years by using the Brigance Parent–Child Interactions Scale (BPCIS) [37] as the assessment tool. The BPCIS is an 18-item parent-report tool that was developed on the basis of the relevant literature; the BPCIS has demonstrated adequate reliability and validity in previous studies [38, 39]. This scale was designed to assess parents’ parenting practices and perceptions of their children (e.g., parents’ responding to their children’s attempts at communication in an encouraging manner and parental confidence in their ability to soothe their children). The aim was to identify positive and problematic parent–child interactions. For analysis purposes, the total score (i.e., the sum of the responses to all 18 items) was used.

#### Covariates

During the early stages of pregnancy, the participating mothers completed a baseline survey to provide parental sociodemographic data, including those related to age, education, and parity. Additionally, we collected data related to each child’s sex at birth, gestational age (in weeks), and birth weight (in g) from the child’s health booklet, which is completed by a health-care provider at the time of a child’s birth at a hospital. Parental depression at 1 year postpartum was assessed using the 10-item Edinburgh Postnatal Depression Scale [40]. The cutoff scores for higher and lower depression levels were 13 and 12, respectively, for mothers [41] and 10 and 9, respectively, for fathers [42].

### Statistical analyses

To analyze the CBCL questionnaire responses at the age of 3 years, we adhered to the recommendations outlined by Achenbach and Rescorla [43] and another previous study [44]. Specifically, raw scores were used to evaluate the children’s behavioral performance. Furthermore, each behavioral outcome across the seven domains of CBCL assessment, along with the aforementioned three additional categories (internalizing, externalizing, and total problems), was examined in each model.

Group-based trajectory modeling (GBTM), a finite mixture model that allows for variations in the shape of trajectories across groups, enabled the determination of a dose-accumulative relationship between screen exposure and developmental outcomes within a longitudinal framework [45]. We employed this approach to determine changes in children’s screen time and mother–child interaction patterns across the ages of 1, 2, and 3 years.

Regarding smart device usage time, the two identified trajectories satisfied the criteria for a suitable parsimonious model, as indicated by the lowest Bayesian information criterion (BIC) of − 2807.26, an average posterior probability of ≥ 0.94 for each group, and odds of correct classification of 117.91 and 7.52 for the two groups categorized as “considerably increased” and “slightly increased” usage, respectively (Supplementary Fig. S2). The model demonstrated good fit for mother–child interactions (BIC = − 1,915.04; average posterior probability = 0.92; odds of correct classification = 38.14 and 7.05 for the two groups of “low” and “high” interaction, respectively; Supplementary Fig. S3). Stata/SE version 17.0 (StataCorp, College Station, TX, USA) and the “traj” procedure [46] were employed for assessments.

All data were analyzed using descriptive statistics by conducting a two-sample *t* test for continuous variables and either the chi-squared test or Fisher’s exact test for categorical variables as appropriate. This approach was used to examine factors associated with multiple smart device trajectories categorized through GBTM, as well as the media content consumed. Moreover, changes in a child’s smart device usage during the ages of 1–3 years were examined using a repeated-measures analysis of variance (ANOVA). Subsequently, multiple linear regression models were employed to examine the association between smart device usage (including screen time trajectories and media content consumption) and the behavioral performance of the children at the age of 3 years. In these multiple linear regression models, candidate variables were selected on the basis of their potential associations with the children’s smart device usage and behavioral outcomes in bivariate analyses (*p* < 0.20) or on the basis of their previous identification as potential confounding factors in related studies [16, 24, 25, 29–31]. Considering that the missing value percentages among covariates ranged from 0.35 to 7.29%, multiple imputation was employed to address these gaps. However, because the results obtained from both regular multiple regression and the multiple imputation approaches were relatively consistent, the findings from the regular multiple regression models were reported to maintain alignment with the observed data. Finally, subgroup analysis stratified by mother–child interactions was conducted because interaction terms for the variables with the children’s smart device usage indicated statistical significance.

All statistical analyses were performed using Stata/SE, and a two-sided *p-value* of < 0.05 was considered to indicate statistical significance.

## Results

Table 1 presents the distribution of the characteristics of the participating mothers, fathers, and children in relation to screen time trajectory and communication content. A high proportion of children with extensive screen time usage had mothers who were unemployed (*p* = 0.02) and fathers with low educational levels (*p* = 0.002). Additionally, compared with mothers and fathers of children whose screen time slightly increased, those of children whose screen time considerably increased exhibited increases in personal smart device usage (*p* = 0.03 and 0.02, respectively). Moreover, children with higher exposure to communication content tended to have younger and more multiparous mothers (*p* < 0.001). The distribution of sociodemographic characteristics among the participating mothers, fathers, and children in relation to screen time trajectory and media content exposure is further illustrated in Tables 1 and 2.

**Table 1:** Distribution of parent and child characteristics, stratified by screen time trajectory and content exposure

Variable	Screen time trajectory (*n* = 277)				Communication content exposure (*n* = 241)		
	Total sample ^a^	Slightly increased (*n* = 246) n (%)	Considerably increased (*n* = 31) n (%)	p-value	Low use (*n* = 142) n (%)	High use (*n* = 99) n (%)	p-value
*Maternal characteristics*							
Age (in years, mean ± SD)	274	33.51 ± 4.06	33.43 ± 4.55	0.46	33.81 ± 4.17	32.84 ± 4.00	0.04
Educational level	276						
High school or lower		71 (28.98)	4 (12.90)	0.08^b^	9 (6.34)	3 (3.06)	0.20 ^b^
Undergraduate or higher		174 (71.02)	27 (87.10)		133 (93.66)	95 (96.94)	
Employment status	275						
Unemployed		26 (10.66)	8 (25.81)	0.02	17 (12.06)	10 (10.10)	0.64
Employed		218 (89.34)	23 (74.19)		124 (87.94)	89 (89.90)	
Depression level	258						
Low		195 (85.15)	22 (75.86)	0.20	113 (84.96)	78 (85.71)	0.88
High		34 (14.85)	(24.14)		20 (15.04)	13 (14.29)	
Smart device use (mean ± SD)	119	180.25 ± 122.61	278.86 ± 157.98	0.003	183.61 ± 122.50	197.21 ± 132.82	0.30
Mother–child interactions	277						
Low		60 (24.39)	5 (16.13)	0.30	36 (25.35)	20 (20.20)	0.35
High		186 (75.61)	26 (83.87)		106 (74.65)	79 (79.80)	
*Paternal characteristics*							
Age (in years, mean ± SD)	266	35.27 ± 4.48	35.70 ± 5.80	0.32	35.54 ± 4.84	34.64 ± 4.42	0.08
Educational level	274						
High school or lower		16 (6.58)	7 (22.58)	0.002	14 (9.93)	8 (8.25)	0.66
Undergraduate or higher		227 (93.42)	24 (77.42)		127 (90.07)	89 (91.75)	
Employment status	274						
Unemployed		30 (12.35)	4 (12.90)	0.56 ^b^	18 (12.68)	14 (14.58)	0.67
Employed		213 (87.65)	27 (87.10)		124 (87.32)	82 (85.42)	
Depression level	254						
Low		176 (78.22)	23 (79.31)	0.89	101 (76.52)	72 (80.00)	0.54
High		49 (21.78)	6 (20.69)		31 (23.48)	18 (20.00)	
Smart device use (mean ± SD)	116	169.69 ± 92.55	233.71 ± 193.39	0.02	183.61 ± 122.50	197.21 ± 132.82	0.30
*Child characteristics*							
Parity	277						
Primiparous		154 (62.60)	21 (67.74)	0.58	156 (64.73)	78 (54.93)	< 0.001
Multiparous		92 (37.40)	10 (32.26)		85 (35.27)	64 (45.07)	
Gestational age (in weeks)	261						
<37		20 (8.62)	4 (13.79)	0.27 ^b^	11 (8.09)	9 (9.78)	0.66
≥37		212 (91.38)	25 (86.21)		125 (91.91)	83 (90.22)	
Birth weight (in g)	277						
<2,500		23 (9.35)	3 (9.68)	0.58 ^b^	13 (9.15)	9 (9.09)	0.99
≥2,500		223 (90.65)	28 (90.32)		129 (90.85)	90 (90.91)	
Infant sex	256						
Male		116 (51.10)	13 (44.83)	0.53	72 (54.14)	40 (44.44)	0.16
Female		111 (48.90)	16 (55.17)		61 (45.86)	50 (55.56)	
*CBCL behavioral problems (mean ± SD)*							
Emotional reactivity	276	2.29 ± 2.05	2.06 ± 2.17	0.28	2.22 ± 2.16	2.33 ± 2.04	0.35
Anxiety/depression	277	2.54 ± 2.06	2.26 ± 2.21	0.24	2.64 ± 2.26	2.32 ± 1.85	0.13
Somatic complaints	276	1.62 ± 1.41	1.74 ± 1.71	0.34	1.62 ± 1.51	1.58 ± 1.33	0.42
Withdrawal	277	1.46 ± 1.68	1.68 ± 2.21	0.25	1.52 ± 1.89	1.36 ± 1.63	0.25
Sleep problems	276	3.27 ± 2.53	3.00 ± 2.32	0.29	3.17 ± 2.50	3.29 ± 2.47	0.36
Attention problems	277	2.06 ± 1.60	2.32 ± 1.38	0.19	2.12 ± 1.65	2.04 ± 1.49	0.35
Aggressive behavior	276	8.18 ± 5.88	7.42 ± 5.93	0.25	8.06 ± 6.13	7.72 ± 5.34	0.33
Internalizing behavior	276	7.92 ± 5.58	7.74 ± 6.67	0.43	8.00 ± 6.25	7.60 ± 5.20	0.30
Externalizing behavior	276	10.24 ± 6.93	9.74 ± 7.03	0.35	10.18 ± 7.34	9.77 ± 6.29	0.32
Total	276	30.22 ± 17.62	29.65 ± 21.13	0.43	30.25 ± 19.36	29.06 ± 16.41	0.31

SD: standard deviation. ^a^Total count may vary because of missing values. ^b^Fisher’s exact test; all other values are from chi-squared tests

**Table 2:** Distribution of parent and child characteristics, stratified by educational, game and cartoon content exposure

Variable	Educational content (*n* = 241)				Game and cartoon content (*n* = 243)		
	Total sample ^a^	Low use (*n* = 139) n (%)	High use (*n* = 102) n (%)	p-value	Low use (*n* = 118) n (%)	High use (*n* = 125) n (%)	p-value
*Maternal characteristics*							
Age (in years, mean ± SD)	239	33.72 ± 3.97	32.98 ± 4.32	0.09	33.69 ± 3.76	33.13 ± 4.43	0.15
Educational level	240						
High school or lower		6 (4.35)	7 (6.86)	0.40	7 (5.93)	6 (4.84)	0.71
Undergraduate or higher		132 (95.65)	95 (93.14)		111 (94.07)	118 (95.16)	
Employment status	240						
Unemployed		16 (11.51)	13 (12.87)	0.75	15 (12.71)	14 (11.29)	0.73
Employed		123 (88.49)	88 (87.13)		103 (87.29)	110 (88.71)	
Depression level	224						
Low		114 (85.07)	76 (84.44)	0.90	95 (84.82)	96 (84.96)	0.98
High		20 (14.93)	14 (15.56)		17 (15.18)	17 (15.04)	
Smart device use (mean ± SD)	103	177.22 ± 130.89	215.51 ± 133.05	0.07	164.71 ± 124.13	218.65 ± 134.93	0.02
Mother–child interactions	241						
Low		36 (25.90)	19 (18.63)	0.18	29 (24.58)	26 (20.80)	0.48
High		103 (74.10)	83 (81.37)		89 (75.42)	99 (79.20)	
*Paternal characteristics*							
Age (in years, mean ± SD)	230	35.50 ± 4.40	34.94 ± 5.15	0.19	35.42 ± 4.30	35.08 ± 5.09	0.30
Educational level	238						
High school or lower		10 (7.35)	12 (11.76)	0.25	9 (7.69)	13 (10.57)	0.44
Undergraduate or higher		126 (92.65)	90 (88.24)		108 (92.31)	110 (89.43)	
Employment status	238						
Unemployed		24 (17.52)	8 (7.92)	0.03	19 (16.24)	13 (10.57)	0.20
Employed		113 (82.48)	93 (92.08)		98 (83.76)	110 (89.43)	
Depression level	222						
Low		103 (77.44)	70 (78.65)	0.83	84 (76.36)	90 (79.65)	0.55
High		30 (22.56)	19 (21.35)		26 (23.64)	23 (20.35)	
Smart device use (mean ± SD)	102	189.70 ± 123.88	169.24 ± 105.73	0.19	196.40 ± 141.56	165.38 ± 86.36	0.09
*Child’s characteristics*							
Parity	241						
Primiparous		92 (66.19)	63 (61.76)	0.48	75 (63.56)	82 (65.60)	0.74
Multiparous		47 (33.81)	39 (38.24)		43 (36.44)	43 (34.40)	
Gestational age (in weeks)	228						
< 37		14 (10.53)	6 (6.32)	0.27	12 (10.81)	8 (6.72)	0.27
≥ 37		119 (89.47)	89 (93.68)		99 (89.19)	111 (93.28)	
Birth weight (in g)	241						
< 2,500		15 (10.79)	7 (6.86)	0.30	9 (7.63)	13 (10.40)	0.45
≥ 2,500		124 (89.21)	95 (93.14)		109 (92.37)	112 (89.60)	
Infant sex	223						
Male		62 (47.69)	50 (53.76)	0.37	55 (50.46)	59 (50.86)	0.95
Female		68 (52.31)	43 (46.24)		54 (49.54)	57 (49.14)	
*CBCL behavioral problems (mean ± SD)*							
Emotional reactivity	240	2.32 ± 1.98	2.21 ± 2.28	0.34	2.16 ± 1.88	2.36 ± 2.29	0.23
Anxiety/depression	241	2.45 ± 1.78	2.67 ± 2.47	0.21	2.45 ± 1.82	2.60 ± 2.33	0.29
Somatic complaints	240	1.71 ± 1.42	1.52 ± 1.51	0.17	1.62 ± 1.38	1.62 ± 1.53	0.50
Withdrawal	241	1.51 ± 1.65	1.40 ± 1.95	0.32	1.37 ± 1.61	1.54 ± 1.93	0.24
Sleep problems	240	3.21 ± 2.34	3.26 ± 2.68	0.44	3.03 ± 2.14	3.39 ± 2.75	0.13
Attention problems	241	2.10 ± 1.46	2.11 ± 1.75	0.49	2.01 ± 1.38	2.18 ± 1.75	0.19
Aggressive behavior	240	7.97 ± 5.05	7.99 ± 6.71	0.49	7.97 ± 4.95	7.90 ± 6.50	0.47
Internalizing behavior	240	7.99 ± 5.04	7.81 ± 6.75	0.41	7.62 ± 4.92	8.12 ± 6.53	0.25
Externalizing behavior	240	10.07 ± 6.05	10.10 ± 7.96	0.49	9.97 ± 5.82	10.09 ± 7.79	0.45
Total	240	30.05 ± 15.57	29.90 ± 21.42	0.48	29.35 ± 14.97	30.38 ± 20.81	0.33

SD: standard deviation. ^a^Total count may vary because of missing values

### Descriptive analysis of children’s smart device usage

**Fig. 1:**
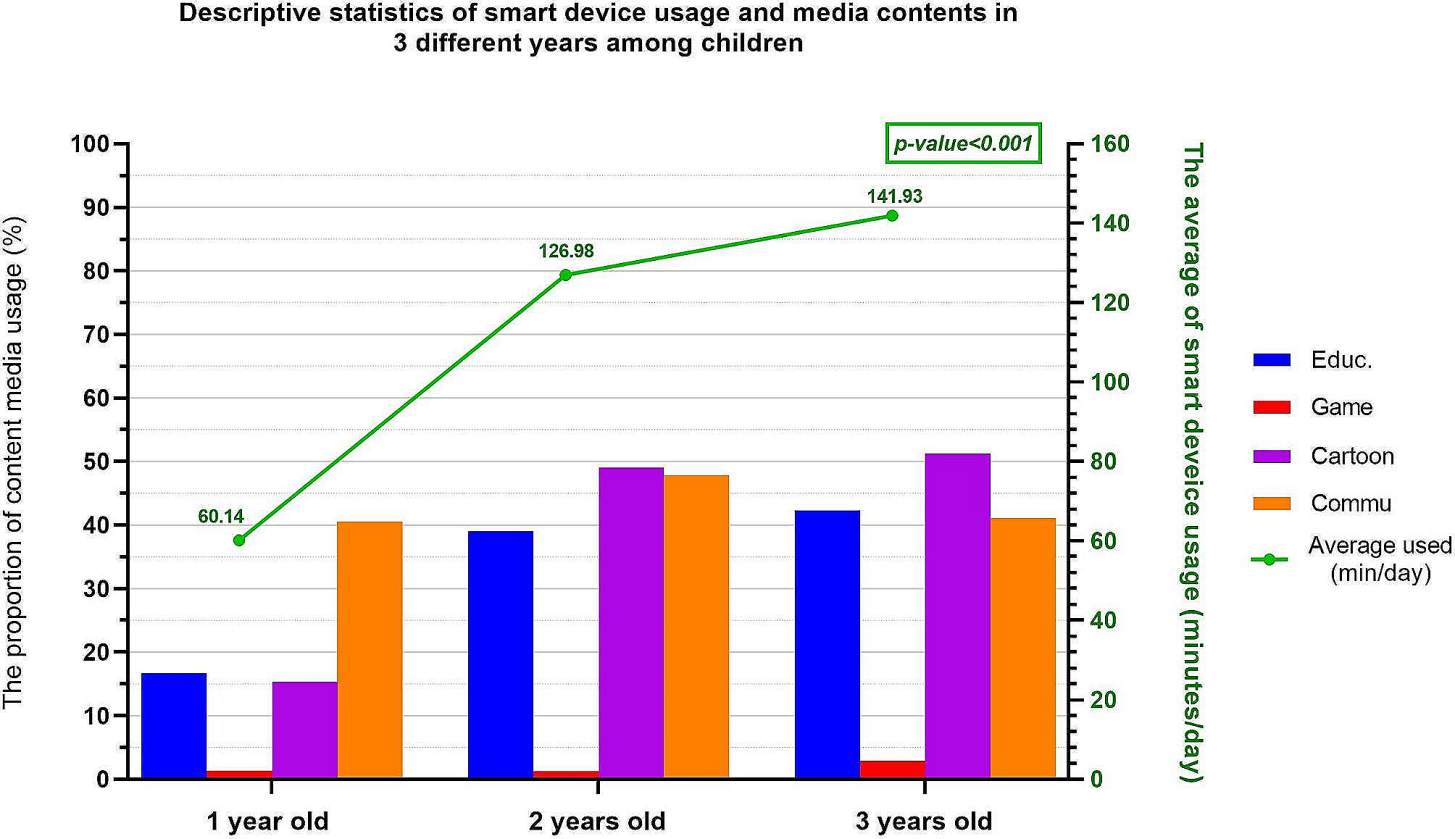
Trends in smart device usage and media consumption from 1 to 3 years of age. ^a^Descriptive statistics detailing shifts in smart device usage time and media content exposure in children aged 1–3 years. On the basis of a 5-point Likert scale, media content exposure variables were dichotomized into two categories: High use (always or often) and low use (sometimes, seldom, or never). Changes in smart device usage over the 3-year period were analyzed using a repeated-measures ANOVA

The results of the repeated-measures ANOVA revealed significant changes in the smart device usage patterns of the children analyzed in this study. The usage significantly increased from 60 min/day at the age of 1 year to 142 min/day at the age of 3 years (*p* < 0.001; Fig. 1). At the age of 1 year, communication content accounted for the highest proportion of smart device usage. However, by the age of 3 years, the proportions of exposure to educational, cartoon, and communication content all exceeded 40%.

### Association between children’s smart device usage and their emotional and behavioral problems

Table 3 presents associations between screen time trajectories and multiple child behavioral outcomes, stratified by mother–child interaction levels. By using multiple linear regression and adjusting for multiple covariates, we observed that between the ages of 1 and 3 years, compared with a slight increase in screen time, a considerable increase in screen time was associated with poorer behavioral outcomes. This finding was particularly evident when mother–child interaction levels were lower. The behavioral outcomes included somatic complaints [adjusted beta coefficient (aβ) = 2.17, 95% confidence interval (CI) = 0.39 to 3.95, *p* = 0.01], withdrawal (aβ = 2.42, 95% CI = 0.15 to 4.69, *p* = 0.04), aggressive behaviors (aβ = 6.53, 95% CI = 0.25 to 12.81, *p* = 0.04), internalizing behaviors (aβ = 8.16, 95% CI = 1.32 to 15.00, *p* = 0.02), externalizing behaviors (aβ = 8.08, 95% CI = 0.67 to 15.50, *p* = 0.03), and total behavioral problems (aβ = 23.46, 95% CI = 2.91 to 44.00, *p* = 0.02). Conversely, in children with higher levels of mother–child interaction, most adverse behavioral problems—including emotional reactivity (aβ = −0.98, 95% CI = − 1.88 to − 0.08, *p* = 0.03), anxiety/depression (aβ = −0.94, 95% CI = − 1.87 to − 0.01, *p* = 0.04), somatic complaints (aβ = −0.82, 95% CI = − 1.41 to − 0.23, *p* = 0.01), aggressive behavior (aβ = −2.82, 95% CI = − 5.32 to − 0.32, *p* = 0.03), internalizing behavior (aβ = −3.26, 95% CI = − 5.66 to − 0.87, *p* = 0.01), and total behavioral problems (aβ = −9.14, 95% CI = − 16.62 to − 1.66, *p* = 0.02)—were associated with higher smart device usage.

**Table 3:** Associations between children’s screen time trajectories and behavioral problems, stratified by mother–child interaction

Behavioral outcome	Mother–child interaction trajectories	
	Low (*n* = 65)	High (*n* = 212)
	aβ (95% CI)^a^	aβ (95% CI)^a^
**Screen time trajectories [considerably vs. slightly increased (reference)]**^**b**^		
Emotional reactivity	1.85 (− 0.54 to 4.23)	**−0.98 (− 1.88 to − 0.08)**
Anxiety/depression	1.72 (− 0.49 to 3.94)	**−0.94 (− 1.87 to − 0.01)**
Somatic complaints	**2.17 (0.39 to 3.95)**	**−0.82 (− 1.41 to − 0.23)**
Withdrawal	**2.42 (0.15 to 4.69)**	−0.52 (− 1.26 to 0.22)
Sleep problems	1.11 (− 1.92 to 4.15)	−0.96 (− 2.07 to 0.16)
Attention problems	1.55 (− 0.20 to 3.30)	0.02 (− 0.70 to 0.74)
Aggressive behavior	**6.53 (0.25 to 12.81)**	**−2.82 (− 5.32 to − 0.32)**
Internalizing behavior	**8.16 (1.32 to 15.00)**	**−3.26 (− 5.66 to − 0.87)**
Externalizing behavior	**8.08 (0.67 to 15.50)**	−2.79 (− 5.75 to 0.16)
Total	**23.46 (2.91 to 44.00)**	**−9.14 (− 16.62 to − 1.66)**

Bold numbers present the estimates in which 95% CI does not include 0

^**a**^ Adjusted for maternal age, maternal educational level, maternal depression level, paternal age, parity, preterm birth, child’s low-birth-weight status, and child’s sex

^b^ The sample sizes were 60 and 5 for the considerably and slightly increased screen time groups, respectively, among those with lower mother–child interaction. Moreover, the sample sizes were 186 and 26 for the two screen time groups, respectively, among those with higher mother–child interaction

### Association between children’s media content and their emotional and behavioral problems

Table 4 presents associations between exposure to various media content types and behavioral problems in children, stratified by levels of mother–child interaction. In children with higher mother–child interaction levels, higher exposure to educational content was associated with fewer somatic complaints (aβ = −0.42, 95% CI = − 0.83 to − 0.01, *p* = 0.02). Conversely, in children with lower levels of mother–child interaction, higher exposure to educational content was associated with more aggressive behaviors (aβ = 4.38, 95% CI = 0.59 to 8.16, *p* = 0.04) and more externalizing behaviors (aβ = 5.19, 95% CI = 0.67 to 9.70, *p* = 0.03). Notably, game and cartoon content exposure also demonstrated considerable effects; lower levels of mother–child interaction were associated with increased exposure to game and cartoon content and adverse outcomes such as increased emotional reactivity (aβ = 1.37, 95% CI = 0.01 to 2.73, *p* = 0.04), reactivity related to anxiety/depression (aβ = 1.54, 95% CI = 0.39 to 2.70, *p* = 0.01), somatic complaints (aβ = 1.24, 95% CI = 0.27 to 2.22, *p* = 0.01), internalizing behaviors (aβ = 5.74, 95% CI = 0.39 to 2.69, *p* = 0.01), and total behavioral problems (aβ = 13.14, 95% CI = 1.64 to 24.63, *p* = 0.03). Conversely, these negative effects were not observed in children with higher mother–child interaction levels. Finally, an increase in exposure to communication content was associated with increased withdrawal behaviors (aβ = 2.04, 95% CI = 0.18 to 3.90, *p* = 0.03), particularly in children with lower mother–child interaction levels.

**Table 4:** Associations between children’s media content exposure and behavioral problems stratified by mother–child interaction

Behavioral outcome	Educational content^a^		Game and cartoon content^a^		Communication content^a^	
	Mother–child interaction trajectories		Mother–child interaction trajectories		Mother–child interaction trajectories	
	Low (*n* = 55)	High (*n* = 186)	Low (*n* = 55)	High (*n* = 188)	Low (*n* = 56)	High (*n* = 185)
	aβ (95% CI)^b^	aβ (95% CI)^b^	aβ (95% CI)^b^	aβ (95% CI)^b^	aβ (95% CI)^b^	aβ (95% CI)^b^
**Media content exposure [high vs. low use (reference)]**						
Emotional reactivity	1.16 (− 0.37 to 2.69)	−0.26 (− 0.91 to 0.38)	**1.37 (0.01 to 2.73)**	0.04 (− 0.60 to 0.67)	−0.26 (− 2.28 to 1.76)	0.01 (− 0.64 to 0.67)
Anxiety/depression	0.23 (− 1.17 to 1.63)	0.17 (− 0.50 to 0.85)	**1.54 (0.39 to 2.70)**	−0.11 (− 0.77 to 0.54)	0.77 (− 1.03 to 2.57)	−0.32 (− 1.00 to 0.35)
Somatic complaints	0.13 (− 1.04 to 1.30)	**−0.42 (− 0.83 to − 0.01)**	**1.24 (0.27 to 2.22)**	−0.3 (− 0.71 to 0.11)	0.15 (− 1.34 to 1.65)	0.13 (− 0.30 to 0.55)
Withdrawal	0.43 (− 1.12 to 1.98)	−0.23 (− 0.76 to 0.29)	**1.58 (0.28 to 2.88)**	−0.15 (− 0.66 to 0.37)	**2.04 (0.18 to 3.90)**	−0.07 (− 0.60 to 0.46)
Sleep problems	1.25 (− 0.72 to 3.22)	−0.04 (− 0.81 to 0.72)	1.5 (− 0.25 to 3.25)	−0.02 (− 0.77 to 0.73)	0.73 (− 1.83 to 3.30)	0.07 (− 0.70 to 0.84)
Attention problems	0.81 (− 0.30 to 1.92)	0.00 (− 0.51 to 0.50)	0.43 (− 0.59 to 1.45)	0.26 (− 0.23 to 0.75)	−0.54 (− 1.97 to 0.89)	0.14 (− 0.37 to 0.64)
Aggressive behavior	**4.38 (0.59 to 8.16)**	−0.89 (− 2.56 to 0.78)	2.79 (− 0.77 to 6.35)	−0.99 (− 2.63 to 0.64)	−1.75 (− 6.89 to 3.39)	0.29 (− 1.39 to 1.97)
Internalizing behavior	1.95 (− 2.61 to 6.52)	−0.74 (− 2.47 to 0.99)	**5.74 (2.06 to 9.42)**	−0.52 (− 2.22 to 1.18)	2.7 (− 3.16 to 8.57)	−0.25 (− 2.00 to 1.49)
Externalizing behavior	**5.19 (0.67 to 9.70)**	−0.89 (− 2.89 to 1.12)	3.22 (− 1.04 to 7.48)	−0.73 (− 2.70 to 1.23)	−2.29 (− 8.41 to 3.82)	0.43 (− 1.59 to 2.45)
Total	9.77 (− 3.45 to 22.99)	−2.06 (− 7.33 to 3.20)	**13.14 (1.64 to 24.63)**	−1.8 (− 6.96 to 3.36)	2.08 (− 15.34 to 19.50)	0.5 (− 4.80 to 5.81)

Bold numbers present the estimates in which 95% CI does not include 0

^a^ Total count may vary because of missing values

^**b**^Adjusted for maternal age, maternal educational level, maternal depression level, paternal age, parity, preterm birth, child’s low birth weight status, and child’s sex

## Discussion

To the best of our knowledge, this study is the first longitudinal study to investigate the effects of smart device usage and media content exposure on the behavioral development of toddlers, with particular focus on the moderating effect of mother–child interaction. Our findings indicate an association between a considerable increase in the screen time trajectories of children from ages 1–3 years and various behavioral problems by the age of 3 years. In addition, this association extended beyond overall screen time to include specific media content, such as games and cartoon media. Notably, these adverse effects were either ameliorated or absent in children with higher mother–child interaction levels, underscoring the vital role of such interactions in moderating the negative effects of smart device usage patterns on young children.

The present results contribute to a limited body of research regarding the effects of early smart device usage on the behavioral patterns of toddlers, particularly internalizing, externalizing, and overall behavioral problems. Consistent with prior research [47, 48], our findings suggest that extensive smart device usage in early childhood is associated with adverse behavioral outcomes, including internalizing behaviors, externalizing behaviors, somatic complaints, social withdrawal, and aggression. Notably, these negative effects were more pronounced in situations characterized by lower levels of mother–child interaction, indicating that such interactions play a crucial role in either mitigating or eliminating behavioral challenges. By contrast, higher levels of mother–child interaction appeared to ameliorate the negative impacts of smart device usage—particularly in relation to anxiety/depression, somatic complaints, and aggression—or render them nonexistent, as observed in relation to withdrawal and externalizing behaviors. Overall, this study addresses a gap in the literature by empirically demonstrating the significant role of parent–child interactions in alleviating the adverse effects of screen time on toddlers’ behavior, thereby corroborating previous suggestions [16].

Although research regarding the impact of smart device media content on toddlers is limited, the present findings corroborate those of a study examining the impact of traditional media [24]. Specifically, toddlers exposed to more educational content within environments with higher levels of parent–child interaction had significantly fewer somatic complaints. Conversely, increased exposure to educational content in settings with lower levels of parent–child interaction was associated with more aggressive behavior in children. Notably, exposure to educational content coupled with warm and supportive parenting was associated with enhanced executive functional development in young children [24]. By contrast, increased exposure to game and cartoon content was associated with an increase in internalizing problems—including anxiety/depression, somatic complaints, and withdrawal—among toddlers, particularly among those experiencing poorer mother–child interactions. Nevertheless, no such associations were observed among toddlers with higher levels of mother–child interaction. Although previous studies have reported negative effects of gaming consoles on children’s behavior [29, 47], our study highlights the importance of mother–child interaction, thereby providing valuable insights into this area of research. However, contrary to previous findings highlighting the beneficial effects of video chatting [49], we observed that toddlers with greater exposure to communication content, particularly those with lower levels of mother–child interaction, tended to exhibit more withdrawal symptoms; this finding warrants further examination.

To elucidate the mechanisms underlying the present findings, drawing on parenting style theory [22], we emphasize the importance of parental interactions, discipline strategies, communication patterns, and responses to children’s behavior (parenting) in child development [50]. By using the BPCIS, the present study assessed mother–child interactions, focusing on parents’ responses to their children’s attempts at communication in an encouraging manner and parents’ confidence in their ability to provide comfort. These aspects are indicative of parenting behaviors and parental efficacy. Through positive interactions aimed at fostering effective parenting practices [51, 52], mothers can play an active role in setting appropriate screen time limits, guiding their children in navigating digital experiences in a healthy way, and participating in media content engagement with their children [52]. Such maternal involvement can contribute to a reduction in screen time and guide children toward beneficial smart device use, such as engagement with educational content [17]. In addition, enhanced mother–child interactions may promote other healthy activities, such as socialization and participation in physical activities, as alternatives to prolonged smart device usage [53]. Furthermore, warm and responsive parenting, characterized by the avoidance of harsh punishments and the allowance of child autonomy with authoritative yet appropriate boundaries [22], is a key feature of positive mother–child interactions. Such interactions are essential for the development of self-regulation in young children [54]. Conversely, children experiencing low levels of self-regulation due to poor mother–child interactions may encounter challenges related to attentional control. In summary, the immediate rewards offered by smart devices frequently foster a strong attachment to such devices, presenting a challenge to children in shifting their focus away from screens in order to engage in other, more beneficial activities during this critical developmental phase [47].

The surge in smart device usage among toddlers in recent years is a major concern, primarily because portable, handheld smart devices are often used by parents as a distraction or reward or as a means of parental respite [26]. Our study revealed a notable increase in daily smart device usage among toddlers, from 60 min at the age of 1 year to 142 min at the age of 3 years, thereby exceeding the recommended screen time for toddlers [4]. By the age of 3 years, the analyzed children were frequently immersed in educational, cartoon, and app content—a trend consistent with the findings of Radesky et al., who also highlighted widespread smart device usage [18]. The interactive functionalities and immersive experiences [55] offered by smart devices can fuel excessive usage in young children [56]. Furthermore, the emission of blue light from screens may disrupt children’s sleep patterns, potentially affecting their overall health and development [27], particularly if used shortly before bedtime [28]. Finally, excessive smart device usage may limit engagement in beneficial activities, such as face-to-face social interactions [57] and physical activities essential for developing visual motor skills [57].

Our study findings underscore several key implications. First, in the current digital era, positive parent–child interactions can enable healthy adaptation to the inevitable integration of smart devices into the lives of young children. Therefore, in line with the recommendations of the American Academy of Pediatrics Family Media Use Plan [4], we advocate the prioritization of mother–child interactions to enhance parenting practices. This study revealed that promoting activities that enhance mother–child interactions, characterized by warm and responsive parenting, can mitigate the adverse impact of excessive screen time on behavioral development. Positive interactions, such as empathetic communication and frequent conversations with children, can counterbalance the potential harms associated with screen time [24]. For instance, encouraging activities that emphasize talking, listening, reading, teaching, and verbal reassurance can be particularly effective. Additionally, during media consumption, fostering collaborative learning experiences between caregivers and children by introducing collaborative activities or modeling teaching strategies, such as dialogic reading and phonetic exercises, can be beneficial [1, 25]. Moreover, integrating parenting intervention programs [58] into health care and health-care education is imperative for enhancing parental knowledge and competence. Second, our findings highlight the influence of both screen time duration and content quality in the context of young children. Although limiting screen time is crucial, the nature of the content consumed can also influence developmental outcomes. Considering the varying impacts of different content types on the behaviors observed in our study, we recommend a balanced approach when it comes to crafting guidelines and parental guidance that considers both the duration of screen time and the quality of the content consumed. Such a holistic approach could ensure that early screen usage is effectively managed.

The current study had several strengths. First, our longitudinal design enabled the assessment of changes in both smart device usage and mother–child interactions over time. This assessment facilitated a dynamic and comprehensive understanding of evolving trends during the crucial developmental period in children (i.e., from 1 to 3 years of age). Second, our study explored behavioral challenges encountered by 3-year-old children encompassing multiple domains, offering valuable insights with potentially far-reaching implications. These findings are particularly relevant considering the pivotal role of the toddler years in shaping behavioral patterns and long-term habits, along with the current lack of findings related to device usage and screen time during those years. Third, the inclusion of paternal variables such as sociodemographic characteristics and depression levels enhanced the current understanding of family dynamics and their impact on children’s behavioral development and smart device usage.

This study also had several notable limitations. First, our participants were recruited only from medical centers in metropolitan Taipei and primarily comprised women of relatively advanced age and relatively high socioeconomic status. This demographic profile may have limited the generalizability of our findings. Second, the use of self-reporting questionnaires may have introduced biases related to social desirability and recall accuracy, particularly in the assessment of mother–child interactions. Mothers may not always accurately perceive or recall the nuances or problematic aspects of these interactions, and this problem may have led to disparities between perceived and actual interactions [59]. Additionally, maternal mental health problems, such as depression, can influence a mother’s perception and reporting of her child’s behaviors and interactions [60]. Therefore, the potential for shared variance bias—given that exposure, outcomes, and moderation were all reported by the mothers—is a concern that may have led to inflated results. Third, we were unable to differentiate between specific applications used by toddlers, such as YouTube, which may be associated with risk of exposure to age-inappropriate content or advertisements. Many apps claim to be educational but lack sufficient empirical evidence to support this claim [18], posing a challenge to the accurate assessment of their impact on child development. Finally, the attrition rate in our study reached 54%. Although no significant differences were observed between the included and excluded participants regarding parental sociodemographic factors, birth outcomes, or child characteristics, loss to follow-up may have limited our sample size, increased the confidence intervals, or compromised the generalizability of our findings. Thus, although we employed inverse probability weighting to account for missing data, the possibility of attrition bias remains a concern.

## Conclusion

The findings of the present study highlight the crucial role of mother–child interactions in ameliorating the adverse effects of early smart device usage—including the effects of screen time and engagement with media content such as games and cartoons, beginning as early as 1 year of age—on behavioral problems in toddlers. Future related research could objectively assess mother–child interactions and benefit from using applications capable of recording real-life usage data to monitor mobile device usage [18]. Furthermore, longitudinal studies spanning the period from childhood through adolescence are warranted to capture the evolving nature of children’s interactions with media and technology as well as the long-term patterns and consequences of early media exposure. Finally, investigating the role of children’s self-regulation in managing smart device usage and exploring the impact of father–child interactions are essential for obtaining a more comprehensive understanding of related pathways and family dynamics in the digital era.

### Electronic supplementary material

Below is the link to the electronic supplementary material.

Supplementary Material 1

Supplementary Material 2

Supplementary Material 3

Supplementary Material 4

7

Supplementary Material 5

## Data Availability

To access the data, contact the corresponding author. Access can be provided upon approval from the Institutional Review Board (IRB) and adherence to the research collaborative agreement guidelines. This requirement is mandated for our research study by our ethics committee and funders.
