# Supplementary material for: The importance of mother-child interaction on smart device usage and behavior outcomes among toddlers: a longitudinal study
Source: Child Adolesc Psychiatry Ment Health. 2024 Jun 28;18:79. doi: 10.1186/s13034-024-00772-6 (PMC11214231; doi:10.1186/s13034-024-00772-6)
Supplement: Supplementary file 1 — Supplementary Material 1 [file 13034_2024_772_MOESM1_ESM.docx]

**Supplementary Table S1**

Distribution of parent and child characteristics between participants included and not included in the analysis.

| **Variable** | **Included in the analysis**  (**n = 277)** | **Excluded from the analysis ^a^**  **(n = 229)** | ***p-value*** |
| --- | --- | --- | --- |
| **Maternal characteristics** |  |  |  |
| Age (in years, mean ± SD) | 33.40±4.14 | 33.78±4.14 | 0.31 |
| Educational level |  |  |  |
| High school or lower | 262 (94.93) | 213 (93.42) | 0.47 |
| Undergraduate or higher | 14 (5.07) | 15 (6.58) |  |
| Employment status |  |  |  |
| Unemployed | 38 (13.82) | 39 (17.03) | 0.32 |
| Employed | 237 (86.18) | 190 (82.97) |  |
| Depression level |  |  |  |
| Low | 216 (78.55) | 182 (79.48) | 0.80 |
| High | 59 (21.45) | 47 (20.52) |  |
| Smart device use (mean ± SD) | 238.99 ± 192.52 | 247.13 ± 140.03 | 0.40 |
| **Paternal characteristics** |  |  |  |
| Age (in years, mean ± SD) | 35.25 ± 4.69 | 35.39 ± 4.40 | 0.37 |
| Educational level |  |  |  |
| High school or lower | 22 (8.00) | 16 (7.21) | 0.74 |
| Undergraduate or higher | 253 (92.00) | 206 (92.79) |  |
| Employment status |  |  |  |
| Unemployed | 34 (12.32) | 29 (13.12) | 0.79 |
| Employed | 242 (87.68) | 192 (86.88) |  |
| Depression level |  |  |  |
| Low | 248 (91.18) | 204 (90.27) | 0.73 |
| High | 24 (8.82) | 22 (9.73) |  |
| Smart device use (mean ± SD) | 186.06 ± 124.60 | 233.15 ± 187.16 | 0.08 |
| **Child’s characteristics** |  |  |  |
| Parity |  |  |  |
| Primiparous | 176 (63.54) | 153 (66.81) | 0.44 |
| Multiparous | 101 (36.46) | 76 (33.19) |  |
| Gestational age (in weeks) |  |  |  |
| <37 | 25 (9.06) | 25 (11.06) | 0.46 |
| ≥37 | 251 (90.94) | 201 (88.94) |  |
| Birth weight (in g) |  |  |  |
| <2,500 | 27 (9.75) | 22 (9.61) | 0.96 |
| ≥2,500 | 250 (90.25) | 207 (90.39) |  |
| Infant sex |  |  |  |
| Male | 136 (50.00) | 115 (52.04) | 0.65 |
| Female | 136 (50.00) | 106(47.96) |  |

^a^ Of the 506 participants expected to complete the CBCL questionnaire at the child’s age of 3 years, 229 were excluded due to loss of contact after the CBCL questionnaire was sent out.
