# Supplementary figures and images for: The importance of mother-child interaction on smart device usage and behavior outcomes among toddlers: a longitudinal study
Source: Child Adolesc Psychiatry Ment Health. 2024 Jun 28;18:79. doi: 10.1186/s13034-024-00772-6 (PMC11214231; doi:10.1186/s13034-024-00772-6)

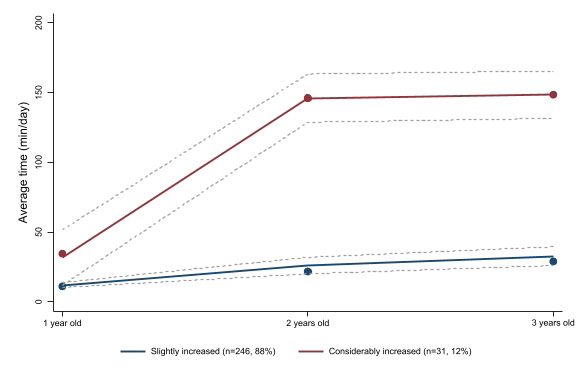

Supplement: Supplementary file 2 — Supplementary Material 2 [file 13034_2024_772_MOESM2_ESM.jpg]

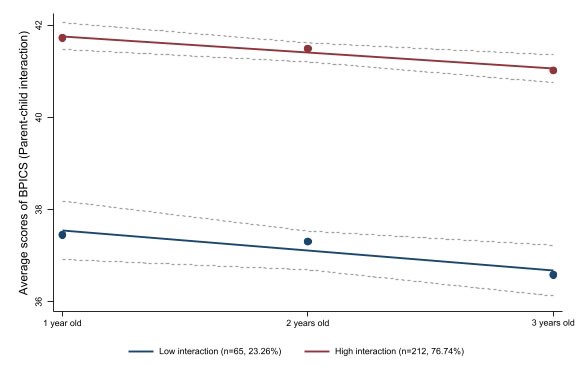

Supplement: Supplementary file 3 — Supplementary Material 3 [file 13034_2024_772_MOESM3_ESM.jpg]

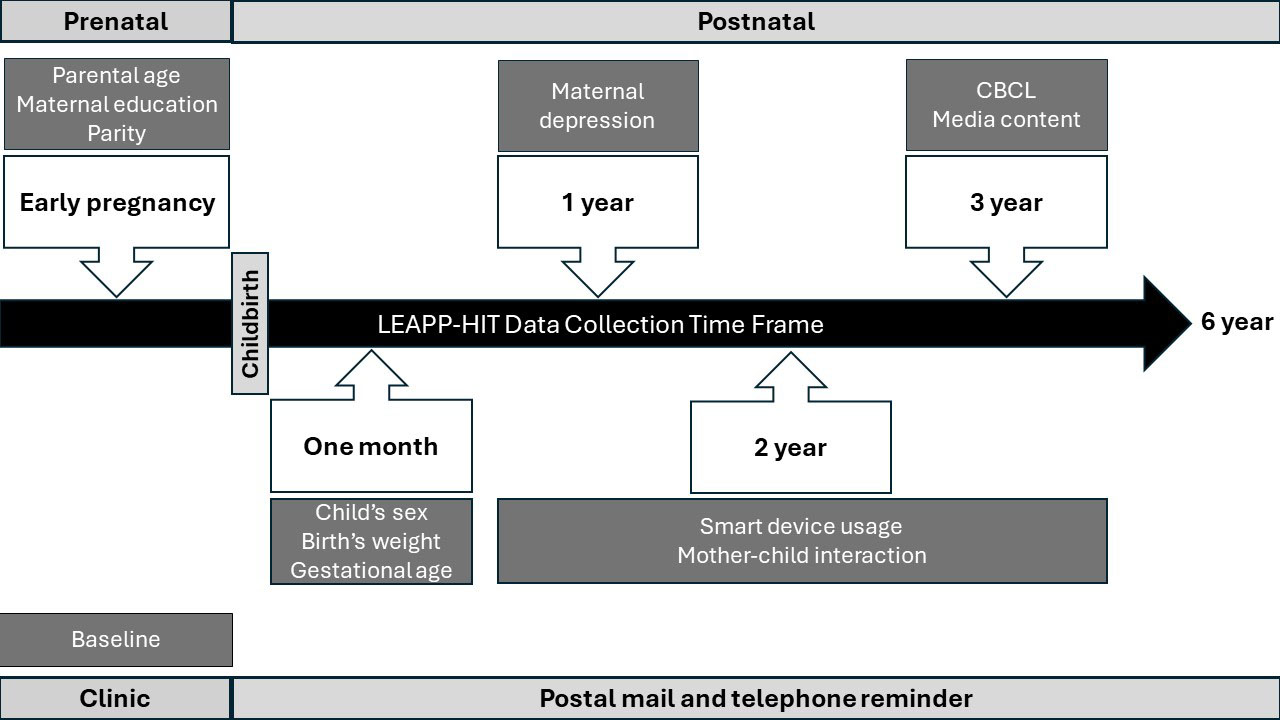

Supplement: Supplementary file 4 — Supplementary Material 4 [file 13034_2024_772_MOESM4_ESM.jpg]
