# Supplementary material for: The importance of mother-child interaction on smart device usage and behavior outcomes among toddlers: a longitudinal study
Source: Child Adolesc Psychiatry Ment Health. 2024 Jun 28;18:79. doi: 10.1186/s13034-024-00772-6 (PMC11214231; doi:10.1186/s13034-024-00772-6)

Supplementary Fig. S1.

Sample recruitment flow.

* Because we introduced the use of the CBCL questionnaire (primary outcome) in November 2019, the available samples included children born between November 2016 and July 2020 who were expected to have a completed questionnaire at the age of 3 years between 2019 and 2023.

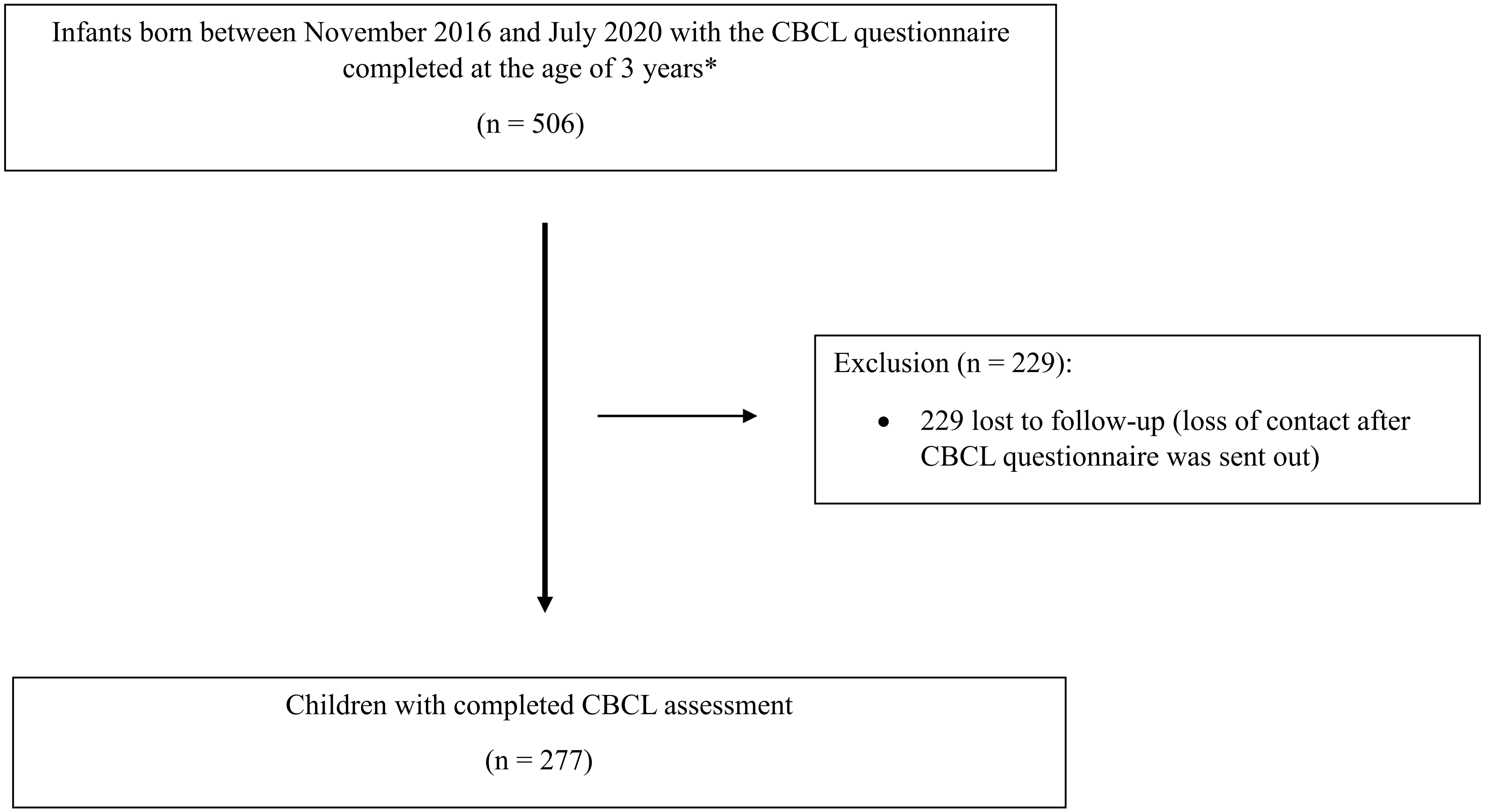

Supplement: Supplementary file 5 — Supplementary Material 5 [file 13034_2024_772_MOESM5_ESM.pdf]
